# Nonlinear Influence of Blending Star and Triblock Copolymers on Morphological and Mechanical Properties of Thermoplastic Elastomers

**DOI:** 10.1002/marc.202500131

**Published:** 2025-05-19

**Authors:** Max G. Schußmann, Leonardo Rocha Dias, Simon Buchheiser, Hermann Nirschl, Jonathan Berson, Manfred Wilhelm, Valerian Hirschberg

**Affiliations:** ^1^ Institute for Chemical Technology and Polymer Chemistry Karlsruhe Institute of Technology (KIT) Engesserstraße 18 76131 Karlsruhe Germany; ^2^ Institute of Mechanical Process Engineering and Mechanics Karlsruhe Institute of Technology (KIT) Am Forum 8 76131 Karlsruhe Germany; ^3^ Institute of Nanotechnology Karlsruhe Institute of Technology (KIT) Am Forum 8 76131 Karlsruhe Germany; ^4^ Institute for Technical Chemistry Technical University Clausthal Arnold‐Sommerfeld‐Str. 4 38678 Clausthal‐Zellerfeld Germany

**Keywords:** blends, block copolymers, complex topologies, mechanical properties, phase separation

## Abstract

To tune the mechanical properties of thermoplastic elastomers, a linear Poly(styrene (S)‐*b*‐isoprene (I)‐*b*‐styrene (S)) triblock copolymer (SIS) is blended with well‐defined symmetric star‐shaped PS‐PI block copolymers with 8 and 15 arms, respectively. The model systems are synthesized via anionic polymerization and grafting‐onto techniques and have a lamellar morphology with the long‐range order *L_0_* for the SIS larger than the stars. Morphological analysis using small angle X‐ray scattering (SAXS) and atom force microscopy (AFM) reveals a nonlinear dependency of *L_0_* as a function of the star content, *ϕ_star_*. For small star contents, *L_0_* of the blends increases above *L_0_* of the individual compounds. The stars show significantly higher mechanical performance than the SIS, by an increased elongation at break, *ε_b_*, by +15% and +38%, and an increased ultimate tensile strength, *σ_UTS_*, by about a factor of 2. For the blends, the mechanical properties highly depend on the star content, and can be fully uncoupled from the ones of the SIS and the star: at small star contents *ε_b_* is significantly improved beyond *ε_b,star_*, but *σ_UTS_* decreases below *σ_UTS,SIS_*. For higher star contents, *σ_UTS_* strongly increases toward *σ_UTS,star_*_,_ whereas *ε_b_* shows a minimum around *ϕ_star_* = 50 wt.%, before increasing to *ε_b,star_*.

## Graphical abstract

To tune the mechanical properties of thermoplastic elastomers, a linear Poly(styrene‐*b*‐isoprene‐*b*‐styrene) triblock copolymer is blended with well‐defined symmetric star‐shaped PS‐PI block copolymers with 8 and 15 arms. Morphological analysis reveals a nonlinear dependency of *L_0_* as a function of the star content and it shows the potential to fully uncouple mechanical properties from the PS/PI volume fraction.

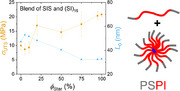

## 1 Introduction

Modifications of polymers have been done since the 1920s by first simple physical blending polystyrene (PS) with a rubbery material to improve mechanical properties.^[^ ^1^ ^]^ Today thermoplastic elastomers (TPE), i.e., materials with rubber‐like elasticity at lower temperatures but thermoplastic‐like processability at higher temperatures are made of block copolymers.^[^ ^2^ ^]^ To generate the mechanical strength of the TPE, phase separation of block copolymers enables the replacement of chemical crosslinking in rubber by physical crosslinking. Commonly linear ABA triblock copolymers are used with a hard block A (high glass transition temperature *T_g_*) and a rubbery block B (low *T_g_)*, i.e., the application temperature of the TPE is below the *T_g_* of the block A, but above the *T_g_* of the block B.^[^ ^3^ ^]^

The phase separation is driven by the repulsion of, unlike polymer segments. The segregation strength in the simple case of block copolymers is described by the product Nχ, where χ is the Flory–Huggins interaction parameter, which decreases ≈1/T, and N is the total degree of polymerization. In a diblock copolymer consisting of two building blocks A and B, the volume composition can be described as

(1) $$f_{A}=\frac{N_{A}\rho_{A}}{N_{A}\rho_{A}+N_{B}\rho_{B}}\;$$

where N is the number of monomers per block, f is the volume fraction of the corresponding monomer and ρ is the respective density. The order–disorder transition, ODT, takes place when Nχ reaches a critical value either by adjusting the total molecular weight of the polymer or by temperature change. The minimum *Nχ* for phase separation to occur is typically at *Nχ* = 10 for linear diblock and *f_A_* = *f_B_* = 0.5^[^ ^4^ ^]^ or *Nχ* = 18 for symmetric ABA triblock copolymers.^[^ ^2^, ^5^ ^]^ In this case self‐assembly leads to a lamellar structure for linear diblock copolymers. For $f_{A}>1/2$, respectively $f_{B}>1/2$, the morphology results in a cylindrical or spherical structure due to interfacial curvature driven by surface tension.^[^ ^6^, ^7^, ^8^ ^]^

For TPEs, a morphology with a continuous soft matrix B with a spherical or hexagonal cylindrical microstructure of A is commonly targeted. For PS−PI block copolymers, χ is at room temperature ≈0.1, requiring therefore N >> 100 for phase separation.^[^ ^9^ ^]^ To avoid a lamellar morphology, less than 35 vol % of the rigid PS component can be copolymerized according to the typical phase diagrams for di‐ and triblock copolymers.^[^ ^8^ ^]^

To improve the mechanical properties of TPEs beyond those of linear ABA triblock copolymers,^[^ ^10^ ^]^ more complex topologies have been investigated including multiblock copolymers,^[^ ^11^, ^12^, ^13^ ^]^ linear low disperse SIS triblock with different shapes of the MWD of the first S block,^[^ ^14^, ^15^, ^16^ ^]^ asymmetric triblocks^[^ ^17^ ^]^ or branched block copolymers with a (miktoarm) star,^[^ ^17^ ^]^ branched comb‐like multigraft copolymers (MGCs)^[^ ^18^, ^19^ ^]^ and pom‐pom^[^ ^20^ ^]^ architectures. It has been shown that branched topologies allow the uncoupling of the volume fraction–morphology relationship of ABA triblock copolymers and enables a significant improvement in the mechanical properties, especially with respect to ultimate tensile stress, *σ_UTS_*, and elongation at break, *ε_b_*.

Whereas ABC terpolymers allow the access of even new morphologies,^[^ ^21^ ^]^ another elegant method to tune morphologies, induce order‐to‐order transitions, and enhance mechanical properties of block copolymers is by blending homo‐ and/or block copolymers of different block length, block number, block order or topology.^[^ ^22^, ^23^, ^24^, ^25^, ^26^, ^27^, ^28^, ^29^ ^]^ Blending an asymmetric ABA triblock copolymer with linear PS homopolymer (hPS) led to a lamellar to hexagonal to lamellar order–order transition with increasing volume fraction of hPS.^[^ ^25^ ^]^ For the blends of a linear PS with a PS/PI miktoarm star, unexpected continuous PI phases could be obtained even at high *ϕ_PS_*: For a blend with 82 wt.% PS, a new morphology with a continuous PI phase was found, described as a “bricks and mortar mesophase.”^[^ ^30^ ^]^ This resulted in a stiff TPE with high elasticity and high elongation at break. For a blend with *ϕ_PS_* = 97 vol% of PS‐PI mictoarm star with hPS, even a lamellar morphology was obtained.^[^ ^31^ ^]^

Tapered SI diblock blended with (SI)_n_ multiblock copolymers with different block numbers and molecular weights of the blocks allowed to substantial increase the mechanical properties of the SI diblock.^[^ ^32^ ^]^ Blends with large differences in the long‐range order *L_0_* between tapered (SI)_5_ and tapered SI of over *ΔL_0_* = 15 nm started to be partially immiscible, with full immiscibility for *ΔL_0_* > 47 nm, i.e., many small domains of the (SI)_5_ appeared to be immiscible with SI containing large domains, which resulted in a decrease in mechanical performance. For the miscible blends, a nearly linear dependency of the domain spacing as a function of the (SI)_3_ weight content was found, combined with a ≈50‐fold increase of the elongation at break for a 50 wt.% blend of SI/(SI)_3_ compared to the neat SI. This could be explained by a bridging of the micro domain via the multiblock copolymers.

In ABA triblock copolymers, the outer A blocks can be located in either the same domain, so that B dorms a so‐called loop, or in two different domains, so B forms a bridge between the two A domains. Domain bridging is associated with increased mechanical performance compared to loops. For SIS triblock copolymers with a lamellar morphology, only ≈41% of the PI blocks were found to bridge,^[^ ^33^, ^34^ ^]^ whereas in stars with SI arms (S outer, I inner block) and with 9 or more arms nearly all PI blocks are predicted to bridge.^[^ ^35^ ^]^

The objective of this article is to investigate the morphological and mechanical properties of blends of linear SIS triblock copolymer and two (SI)_n_ stars with 8 and 15 arms, respectively, similar *ϕ_PS_*, lamellar morphology, and full miscibility. The two (SI)_n_ stars have significantly higher ultimate tensile stress and elongation at break than the linear SIS, so the question is how the mechanical properties of the SIS can be tuned and improved by the addition of the star‐shaped block copolymers. The results show a nonlinear behavior for the morphological domain size of the blends with an unexpected increase of *L_0_* above *L_0_* of the SIS and the star for small star fractions and a drastic increase in mechanical properties with the addition of even 10 wt.% of (SI)_n_.

## 2 Results and Discussion

### 2.1 Materials and Methods

The stars (SI)_8_ and (SI)_15_ were synthesized as described previously.^[^ ^36^ ^]^ The SIS triblock copolymer was synthesized by sequential monomer addition (styrene, isoprene, styrene), initiated by *s*‐BuLi in cyclohexane as described previously.^[^ ^10^ ^]^ Molecular weights were determined by SEC‐MALLS using for dn/dc the weighted average composition as determined by ^1^H NMR spectroscopy with 0.187 mL g^−1^ for PS and 0.111 mL g^−1^ for PI.^[^ ^20^, ^37^ ^]^ The molecular characteristics of the stars and the SIS are listed in **Table** **1**.

**Table 1:** Overview of the molecular, morphological, and mechanical properties of the linear SIS triblock and stars with SI diblock arms used for the blends.

	$M_{w,arm}$ [kg mol^−1^]	$M_{w,t}$ [kg mol^−1^]	Arm number q	*Đ_t_*	*ϕ_PS_* [vol%]	Bulk Morphology	*L_0_* [nm]	*σ_UTS_* [MPa]	*ε_b_* [%]
SIS	−	129	2	1.07	32	L	47.2	9.9 ± 1.5	862 ± 25
(SI)_8_	55	423	8	1.11	28.5	L	29.7	21 ± 3.8	971 ± 58
(SI)_15_	52	788	15	1.08	26	L	32.7	20.8 ± 0.8	1106 ± 36

**Figure 1:**
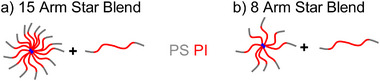
The blends investigated herein are made of a) triblock and 15‐arm star and b) triblock and 8‐arm star.

Blends were prepared by dissolution in THF and stirring overnight. The solution was cast into an aluminum dish and the solvent evaporated slowly over 4 days. Afterward, the films were annealed under vacuum at 120 °C for 4 h. Specimens for SAXS and tensile measurements were cut from the films. Both blend systems are schematically shown in **Figure** **1**.

Stress–strain curves were measured on a Hegewald–Peschke inspect table 10 kN in uniaxial testing at a linear strain rate of 𝜀 = 0.0833 s^−1^ with dog bone specimens (DIN 53 504) at room temperature. Small‐angle X‐ray scattering was conducted on a Xeuss 2.0 Q‐Xoom, Xenocs SA, Grenoble, France, *q* = 0.001 − 4 nm^−1^. Atomic force microscopy (AFM) was done in semicontact mode using a Bruker dimension ICON system equipped with an Opus 160AC‐NA by Mikromasch tip (typical force constant 26 N m^−1^). The AFM samples were prepared on silicon wafers freshly cleaned by snowjet by spin‐coating at 5000 rpm of 0.3 wt.% solutions in toluene, yielding films with a thickness of 15 ± 1 nm.

### 2.2 Morphological Characterization

**Figure 2:**
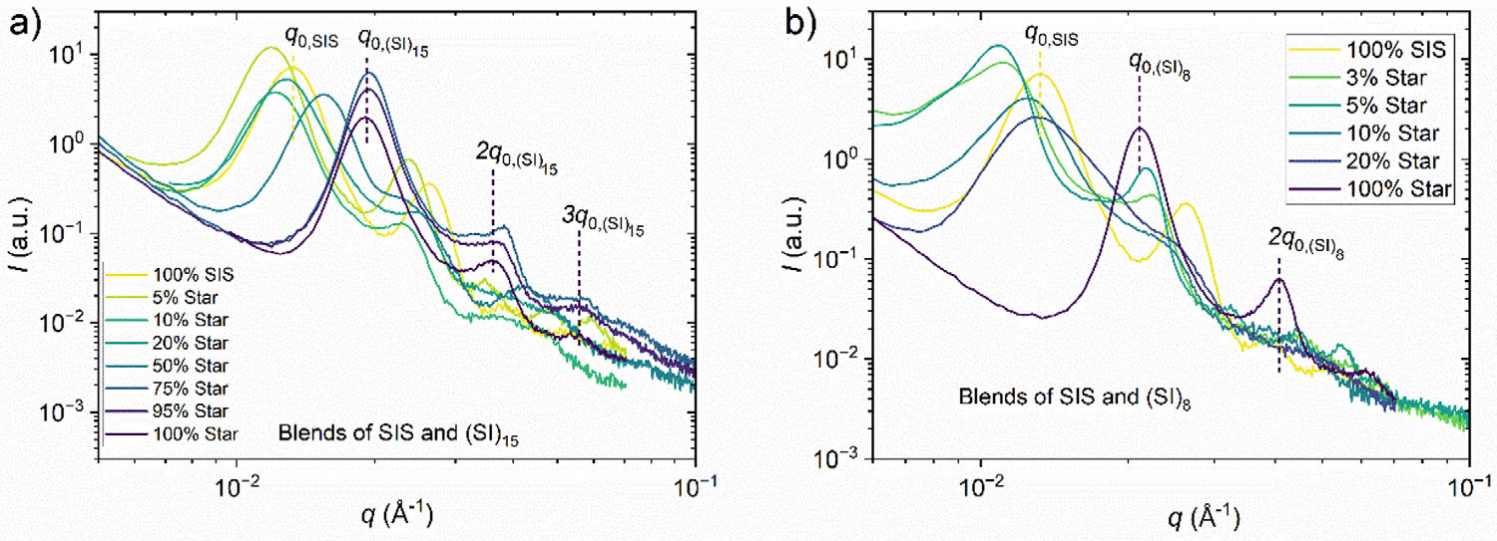
Azimuthally‐averaged SAXS scattering of the blends of the triblock with a) 15‐arm star and b) 8‐arm star. The *q_0_* scattering peaks of neat SIS and star are indicated. For small *ϕ_star_*, the *q_0_* maximum is even shifted to *q*‐values smaller than the neat polymers.

**Figure 3:**
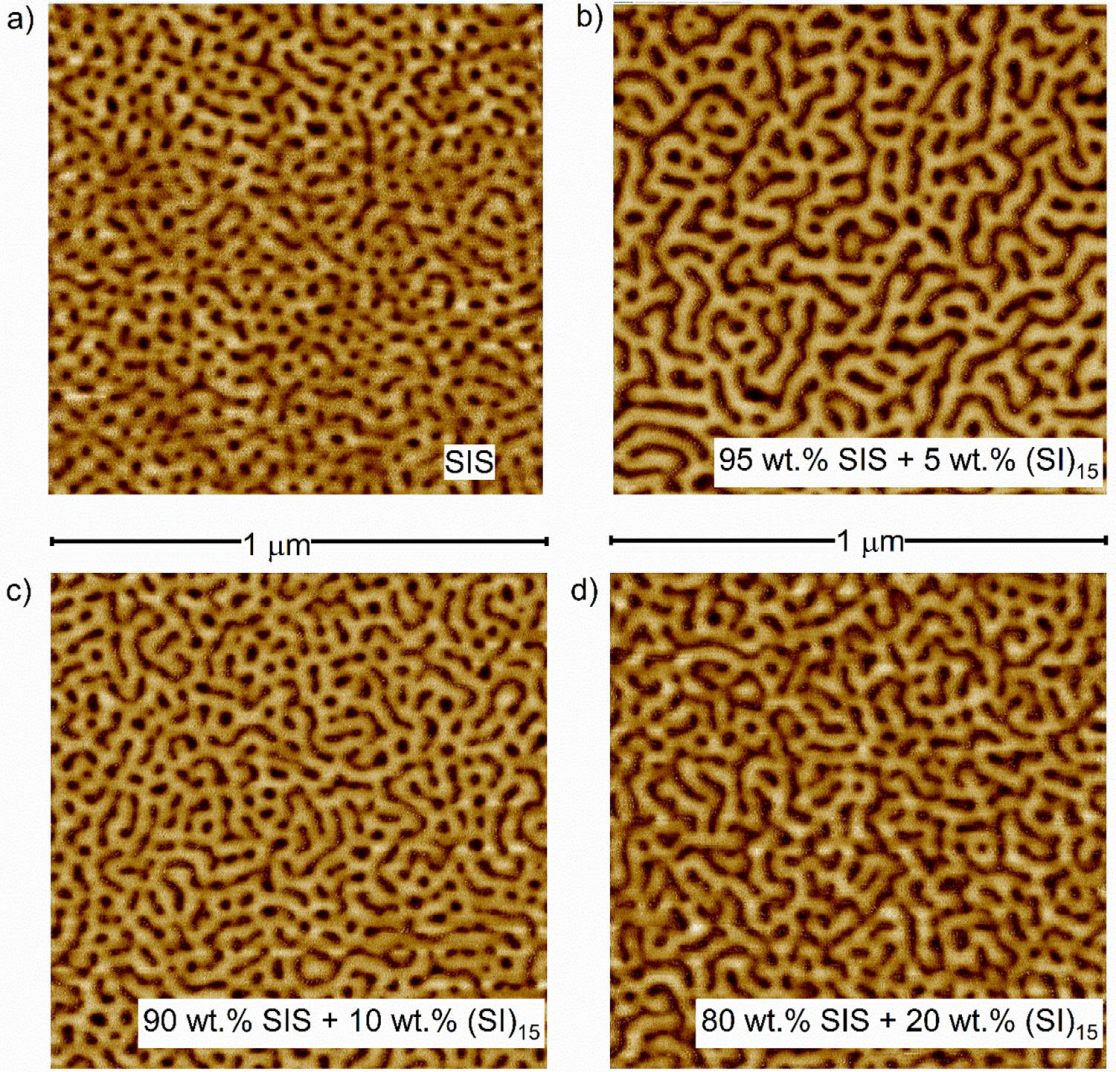
AFM images of the neat SIS and of blends with 5, 10, and 20 wt.% (SI)_15_. The differences between the neat SIS and the blends can be seen clearly as the domain spacing increases. For the blends, the size, or rather the continuous area of the PS domains increases drastically.

To investigate the morphology of the block copolymers and their blends, SAXS and AFM experiments were performed. The SIS and the (SI)_n_ stars have similar *ϕ_PS_* (see Table 1) and as shown by the SAXS and AFM analysis from **Figures** **2** and **3** a lamellar morphology. The SAXS patterns show scattering peaks at *q*_0_, 2*q*_0_, 3*q*_0_, etc., indicating a strong phase separation and a very good long‐range order. SAXS and AFM indicate good miscibility between the linear SIS and the stars. The investigated stars have larger *q_0,_* i.e., smaller long‐range‐order distances *L_0_*, than the SIS with a difference in *L_0_* of *ΔL_0_* = 14.5 nm and 17.5 nm for the 15‐ and the 8‐arm star, respectively.

For the blends of the SIS with the (SI)_15_ in Figure 2a), *q_0_* first decreases below *q_0,SIS_* for (SI)_15_ fractions less than 20 wt.% and then increases to the *q_0_* of the (SI)_15._ A similar trend is observed for the (SI)_8_ blends and the higher order peaks at 2*q_0_* and 3*q_0_*, still indicating a lamellar morphology for all blends. The clearly nonlinear behavior of *L_0_* as a function of *ϕ_Star_* for the SIS/(SI)_n_ blends is also in contrast to the observed behavior of miscible blends of tapered SI and SISISI di‐ and multiblock copolymers, which displayed a nearly linear behavior in the domain spacing with increasing blend content.^[^ ^32^ ^]^

Furthermore, the AFM analysis reveals striking differences between the neat SIS and the SIS/(SI)_n_ blends: compared with the neat SIS the addition of 5 wt.% (SI)_15_ in Figure 3b) not only results in larger domain spacing *L_0_* but also in larger areas of the PS domain. Instead of many small PS domains for the SIS, fewer but larger PS areas can be identified for the SIS/(SI)_15_‐5%. This is also observed for the higher (SI)_15_ weight content, but with again smaller *L_0_*.

### 2.3 Mechanical Testing

**Figure 4:**
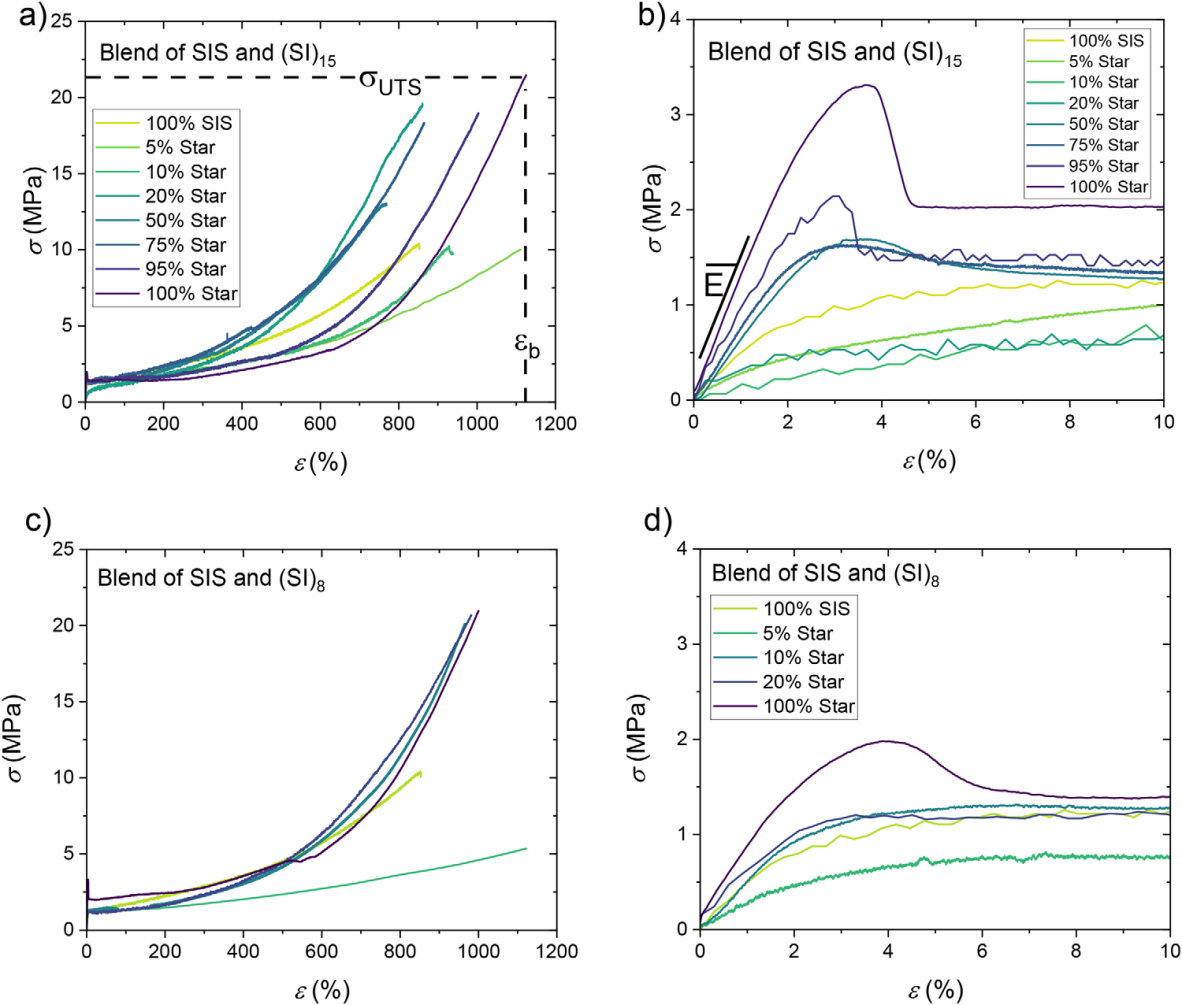
Stress–strain curves of the blends of linear triblock with a) 15 arm star, b) the zoom‐in to small strains, c) 8 arm star, and d) again the zoom‐in to small strains. Tensile tests were conducted at a crosshead speed of 5 mm s^−1^, which corresponds to a linear strain rate of $\dot{\varepsilon }$ = 0.0083 s^−1^.

The stress‐strain curves of the neat SIS, (SI)_15_, (SI)_8,_ and the blends are shown in **Figure** **4** with a zoom for small strains. First, the neat (SI)_15_ and (SI)_8_ clearly outperform the SIS in terms of ultimate tensile stress *σ_UTS_* by about a factor of 2 and reach for both stars around *σ_UTS_* = 21 MPa, which is within the benchmark value for most PS/PI block copolymer model systems, as described in literature overview recently.^[^ ^20^ ^]^ The increase in mechanical performance is also expected from the increase in domain bridging for a star with a high arm number, compared to linear SIS.^[^ ^33^, ^34^, ^35^ ^]^ The elongation of break is increased by ≈20% from *ε_b_* = 860 ± 25% for the SIS to about *ε_b_* > 1000% for the stars. As shown in Figure 4a,b, the neat stars clearly show yielding, which is less pronounced for the SIS.

It is important to note, that tensile tests could not be performed for the blend with 3 wt.% (SI)_8_ in the SIS, since the material was too brittle and the specimen broke during preparation. The mechanical analysis of the blends reveals, that different blend ratios allow tuning the mechanical properties beyond those of the SIS and that the simple expectation of linear addition of the properties of the SIS and the stars do not hold: For the SIS/(SI)_15_ blends with only 5 wt.% of (SI)_15,_ the material becomes very soft with an increase of *ε_b_* = 860 ± 25% for the SIS to *ε_b_* = 1050 ± 71% for the SIS/5%(SI)_15_ blend, but a reduction of *σ_UTS_* by a factor of 2 and 4, compared to the neat SIS and (SI)_8_, respectively. In contrast, the addition of 10 and 20 wt.% of (SI)_15_ to the SIS resulted in a drastic increase in *ε_b_* beyond those of the SIS and *σ_UTS_* nearly increases to the high level of the (SI)_15_. A similar trend was found for the blends with the (SI)_8_ in Figure 4b).

### 2.4 Morphology–Mechanical Property Relationship

**Figure 5:**
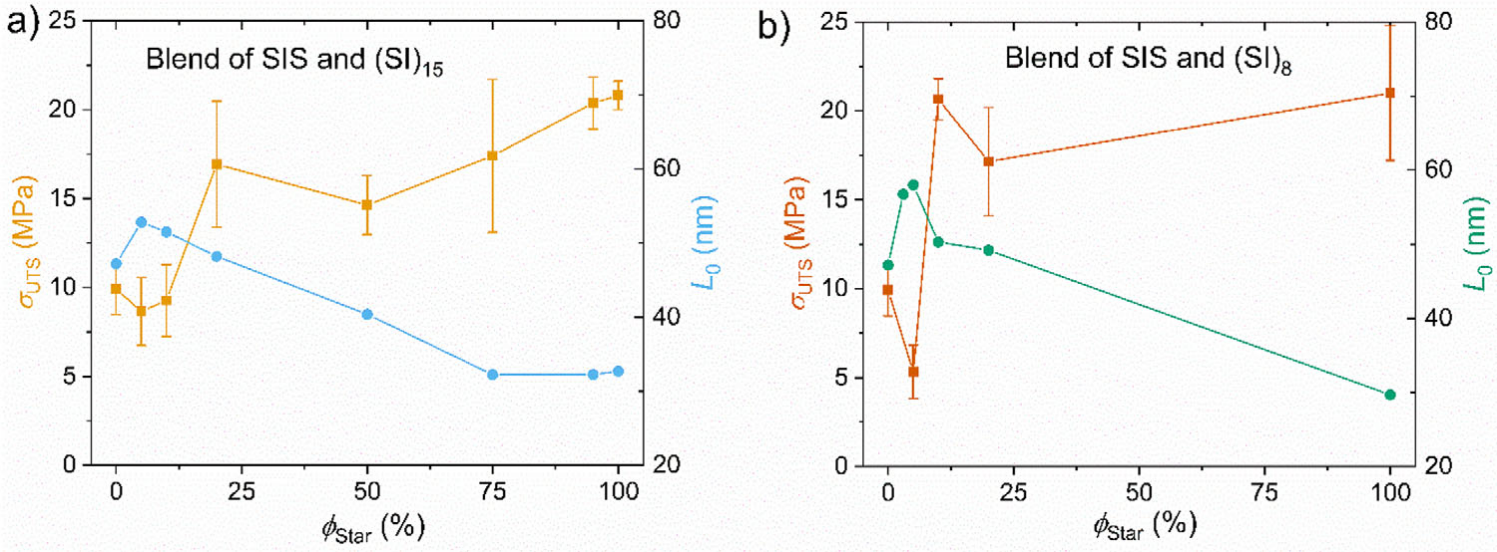
Ultimate tensile strength $\sigma_{UTS}$ of the blends of the SIS with a) the 15‐arm star and b) the 8‐arm star as a function of the star volume fraction on the left *y*‐axis and the domain size $L_{0}$ on the right *y*‐axis.

**Figure 6:**
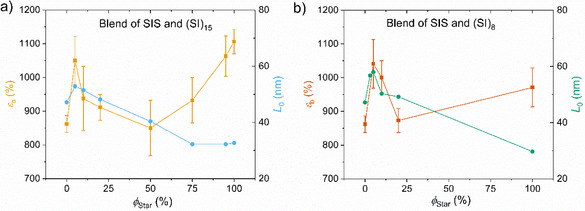
Strain at break $\varepsilon_{b}$ of the blends of the SIS with a) the 15‐arm star and b) the 8‐arm star as a function of the star volume fraction on the left *y*‐axis and the domain size $L_{0}$ on the right *y*‐axis. At *ϕ_star_* < 10 wt.%, L_0_ increases beyond *L_0_* of both, SIS and star, and the elongation at break increases drastically compared with the neat SIS.

**Figure 7:**
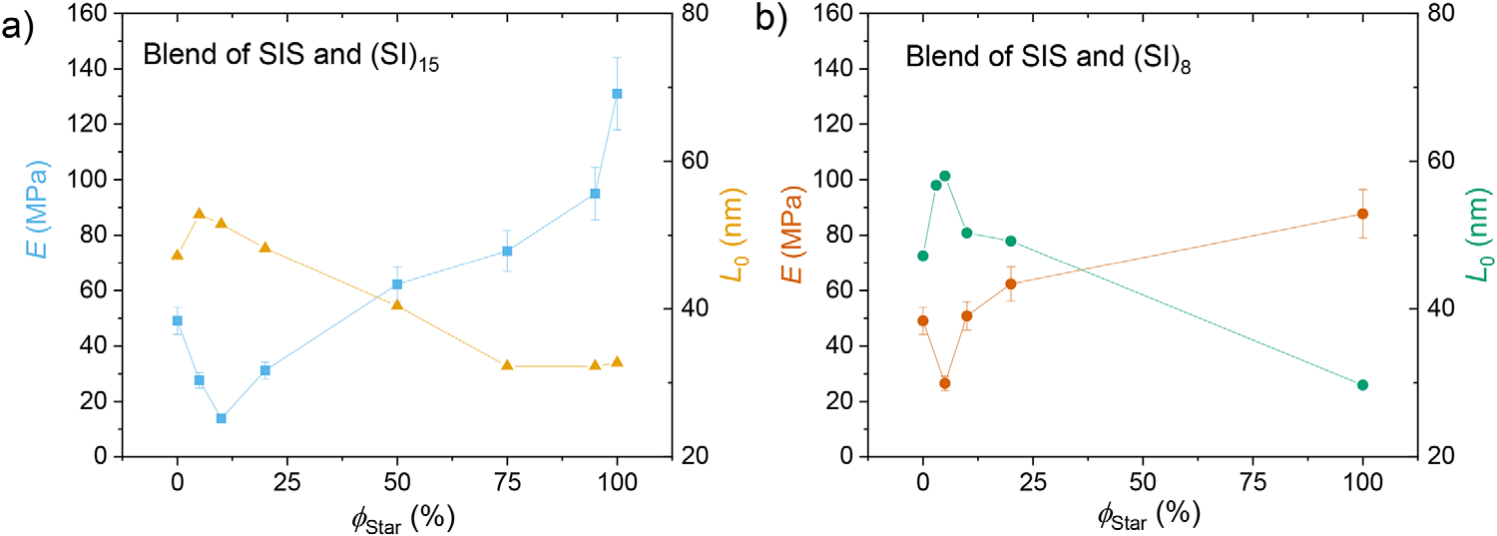
Young's‐Modulus E of the blends of the SIS with a) the 15‐arm star and b) the 8‐arm star as a function of the star volume fraction on the left y‐axis and the domain size $L_{0}$ on the right y‐axis. Similar to $\varepsilon_{b}$, at *ϕ_star_* < 10 wt.%, also Young's modulus decreases drastically below E of the SIS and the star, indicating a softening of the blend.

To establish structure‐property relationships, mechanical key parameters have been analyzed as a function of the star volume fraction *ϕ_star_* together with *L_0_*: in **Figure** **5** the ultimate tensile stress, in **Figure** **6** the elongation at break, and in **Figure** **7** the Young's E modulus. Whereas both stars have higher *σ_UTS_*, *ε_b,_* and Young's modulus, but a smaller *L_0_* than the SIS, the trend for the blends is not simply linear between the properties of the SIS and the stars.

In Figure 5a for the SIS/(SI)_15_ blends, *σ_UTS_* first decreases at *ϕ_star_* = 5 and 10 wt.% when *L_0_* also increases to *L_0_* = 52.8 nm, i.e., beyond *L_0,(SI)15_* = 32.7 nm and *L_0,SIS_* = 47.2 nm. For *ϕ_star_* > 10 wt.%, *σ_UTS_* increases rapidly toward the *σ_UTS_* of the star, with also *L_0_* decreasing below *L_0_* of the SIS and reaching around *ϕ_star_* > 75 wt.% the *L_0_* value of the star. For the SIS/(SI)_8_ blends a similar trend is found at low *ϕ_star_*, where *σ_UTS_* decreases below *σ_UTS_* of the star and correlates with an increased *L_0_*.

In contrast to *σ_UTS_*, as shown in Figure 6a), *ε_b_* increases drastically at low *ϕ_star_*. For the SIS/(SI)_15_ blends, the addition of 5 wt.% (SI)_15_ to the SIS results in an increase from *ε_b_* = 860 ± 25% to *ε_b_* = 1050 ± 71%. With increasing star content, *ε_b_* first decreases again until it reaches around *ϕ_star_* = 50 wt.% a similar *ε_b_* as the SIS, and then increases rapidly toward the *ε_b_* of the neat (SI)_15_. For the (SI)_8_ a similar trend is observed at *ϕ_star_* < 20 wt.%, with a drastic increase in *ε_b_* when also *L_0_* increases above the *L_0_* of the SIS. The Young's modulus is shown in Figure 7a) for the SIS/(SI)_15_ blends, revealing also an unexpected trend at low *ϕ_star_*. For *ϕ_star_* < 20 wt.%, the Young's modulus decreases compared to the Young's modulus of the SIS by up to about a factor of three, i.e., the material gets significantly softer, as also indicated by *ε_b_*. For *ϕ_star_* > 20 wt.%, Young's modulus increases until it finally reaches the value of the neat star. For the SIS/(SI)_8_ blends, a similar trend for Young's modulus as a function of *ϕ_star_* is found.

For *ϕ_star_* < 20 wt.%, the softening of the material quantified by increased *ε_b_* but drastically decreased *σ_UTS_* and *E* as well as the increased *L_0_* indicates reduced domain bridging on the molecular level and increased domain looping of the block copolymer chains. In contrast, it indicates that at high *ϕ_star_* domain bridging is significantly increased compared to domain looping for the pure SIS, as predicted by self‐consistent field theory.^[^ ^33^, ^35^ ^]^

The analysis of the mechanical properties (*σ_UTS_*, *ε_b_* and Young's E modulus) as a function of *ϕ_star_* reveals a complex structure‐property relationship and blending of a SIS and a star‐shaped SI block copolymer allows to tune the material properties beyond the one of the SIS and the star. In particular the unexpected morphological and mechanical behavior of the blends having *ϕ_star_* < 20 wt.% is striking. The addition of *ϕ_star_* < 20 wt.% leads to an increase in *L_0_* beyond those of the SIS and the star, which highly impacts the mechanical properties and leads to a softer material than its individual blend components with lower Young's modulus and ultimate tensile stress, but therefore a significantly increased elongation at break. At *ϕ_star_* > 20 wt.%, the ultimate tensile stress increases drastically compared to the SIS, whereas *ε_b_* and *E* have similar values as the SIS and then increase further to one of the neat stars. The blending of the linear SIS and the (SI)_n_ consequently allows to fully uncouple *L_0_*, *σ_UTS_*_,_ *ε_b,_* and Young's modulus and tunes the desired mechanical properties.

## 3 Conclusion

To tune the mechanical properties of thermoplastic elastomers, a linear Polystyrene (PS)‐polyisoprene (PI)‐polystyrene (PS) triblock copolymer (SIS) is blended with well‐defined star‐shaped PS‐PI block copolymers with 8 and 15 arms, respectively. The low‐disperse model systems have a PS content ≈30 vol% and were synthesized via anionic polymerization and grafting‐onto techniques. The SIS and the stars have a lamellar morphology with the long‐range order *L_0_* for the SIS larger than the stars and are fully miscible with each other as shown by the morphological analysis done with small angle X‐ray scattering (SAXS) and atom force microscopy (AFM). The results reveal a nonlinear trend of *L_0_* as a function of the star content, *ϕ_star_*, with an unexpected increase of *L_0_* above *L_0_* of the SIS and stars for small star contents. The stars have a significantly higher mechanical performance than the SIS, by an increased elongation at break, *ε_b_*, by +15% and +38%, and ultimate tensile strength, *σ_UTS_*, by about a factor of 2. For the blends, the morphological and mechanical properties highly depend on the star content, and can be fully uncoupled from the ones of the SIS and the star: at small star content, *L_0_* of the blends is larger than *L_0_* of the SIS or the stars and *ε_b_* significantly increases beyond *ε_b,star_*, but *σ_UTS_* decreases below *σ_UTS,SIS_*, indicating reduced domain bridging. For higher star contents, *σ_UTS_* strongly increases toward *σ_UTS,star_*_,_ whereas *ε_b_* shows a minimum around *ϕ_star_* = 50 wt.%, before increasing to *ε_b,star_*. This shows the potential to fully uncouple and tune morphological and mechanical properties from molecular characteristics and the PS/PI volume fraction by blending block copolymers of different complex architectures.

Future dielectric spectroscopic investigations of the domain bridging/looping of complex block copolymer blends would be of great interest.

## Conflict of Interest

The authors declare no conflict of interest.

## Data Availability

The data that support the findings of this study are available from the corresponding author upon reasonable request.
